# *Polypodium vulgare* L. (*Polypodiaceae*) as a Source of Bioactive Compounds: Polyphenolic Profile, Cytotoxicity and Cytoprotective Properties in Different Cell Lines

**DOI:** 10.3389/fphar.2021.727528

**Published:** 2021-09-16

**Authors:** Adrià Farràs, Montserrat Mitjans, Filippo Maggi, Giovanni Caprioli, María Pilar Vinardell, Víctor López

**Affiliations:** ^1^ Department of Biochemistry and Physiology, Faculty of Pharmacy and Food Sciences, Universitat de Barcelona, Barcelona, Spain; ^2^ Department of Pharmacy, Faculty of Health Sciences, Universidad San Jorge, Zaragoza, Spain; ^3^ School of Pharmacy, Università di Camerino, Camerino, Italy; ^4^ Instituto Agroalimentario de Aragón-IA2, CITA-Universidad de Zaragoza, Zaragoza, Spain

**Keywords:** cytoprotection, cytotoxicity, polyphenols, polypody, ferns, medicinal plants

## Abstract

Pteridophytes, represented by ferns and allies, are an important phytogenetic bridge between lower and higher plants. Ferns have evolved independently of any other species in the plant kingdom being its secondary metabolism a reservoir of phytochemicals characteristic of this taxon. The study of the potential uses of *Polypodium vulgare* L. (*Polypodiaceae*) as medicinal plant has increased in recent years particularly when in 2008 the European Medicines Agency published a monograph about the rhizome of this species. Our objective is to provide scientific knowledge on the polar constituents extracted from the fronds of *P. vulgare*, one of the main ferns of European distribution, to contribute to the validation of certain traditional uses. Specifically, we have characterized the methanolic extract of *P. vulgare* fronds (PVM) by HPLC-DAD and investigated its potential cytotoxicity, phototoxicity, ROS production and protective effects against oxidative stress by using *in vitro* methods. The 3T3, HaCaT, HeLa, HepG2, MCF-7 and A549 were the cell lines used to evaluate the possible cytotoxic behaviour of the PVM. HPLC-DAD was utilized to validate the polyphenolic profile of the extract. H_2_O_2_ and UVA were the prooxidant agents to induce oxidative stress by different conditions in 3T3 and HaCaT cell lines. Antioxidant activity of *in vitro* PVM in 3T3 and HaCaT cell lines was evaluated by ROS assay. Our results demonstrate that PVM contains significant amounts of shikimic acid together with caffeoylquinic acid derivatives and flavonoids such as epicatechin and catechin; PVM is not cytotoxic at physiological concentrations against the different cell lines, showing cytoprotective and cellular repair activity in 3T3 fibroblast cells. This biological activity could be attributed to the high content of polyphenolic compounds. The fronds of the *P. vulgare* are a source of polyphenolic compounds, which can be responsible for certain traditional uses like wound healing properties. In the present work, fronds of the common polypody are positioned as a candidate for pharmaceutical applications based on traditional medicine uses but also as potential food ingredients due to lack of toxicity at physiological concentrations.

## Introduction

Oxidative stress is characterized by an imbalance between pro-oxidant agents and the antioxidant defence system. According to its origin, this antioxidant system is classified as endogenous or exogenous (Addor, 2017). The inability to maintain an adequate redox state, either due to excess production of free radicals or an alteration of the antioxidant system, triggers oxidative damage that affects fundamental biological structures (Willcox et al., 2004). In this sense, studies have associated oxidative stress with the development of different metabolic diseases (Cásedas et al., 2016; He et al., 2017). Nowadays, antioxidant is defined as “*any substance that delays, prevents, or removes oxidative damage to a target molecule”* (Burton and Ingold, 2015).

Numerous investigations have also established the link between sun exposure and skin alterations (Evans and Johnson, 2010; Kimlin and Guo, 2012). Today it is well documented that ultraviolet radiation affects animals in different causes, among which oxidative stress, inflammation, erythema, breakdown of the extracellular matrix, wrinkling and skin cancer. But the main effect of this ultraviolet irradiation is the increase in oxidative stress caused by the increase in ROS, which can lead to an imbalance in the endogenous antioxidant system (Gegotek et al., 2020). Ultraviolet irradiation is also the main etiologic factor in the development of skin cancers (Rundle et al., 2020). Phytochemicals can modulate the behaviour of tumour cells by acting on different pathways of molecular signalling such as exogenous antioxidant system. As examples of these pathways are the topoisomerase inhibition (genistein), kinase inhibition (apigenin) and modulation of multidrug resistance (2′,4′,6′-tri*OH*-chalcone), among others (Ren et al., 2003). The doxorubicin, paclitaxel, vinblastine, etoposide, irinotecan, gemcitabine, and methotrexate are medically successful in anticancer therapy, for their security and efficacy, which are part of the list of anticancer agents provided or inspired by nature in recent years (Newman and Cragg, 2012; Silva et al., 2019). In addition, polyphenols, as flavonoids for their safety and accessibility, can also be key dietary molecules for cancer treatment and prevention respectively (Asensi et al., 2011; Shukla et al., 2014; Alvarado-Sansininea et al., 2018).

Ethnopharmacological investigations on traditional Chinese medicine have reported the therapeutic uses of ferns in current medicine. For that purpose, different bioassays were performed as for example antioxidant (*Dryoathyrium boryanum* (Willd.) Ching (*Athyriaceae* family) (Cao et al., 2013)), acetycholinesterase inhibition (*Stenochlaena palustres* (Burm. f.) Bedd. (*Blechnaceae* family) (Chear et al., 2016)), tyrosinase inhibition (*Asplenium adiantum-nigrum* L. (*Aspleniaceae* family) (Farràs et al., 2019)) and anti-tumour activity (*Davallia cylindrica* Ching (*Davalliaceae* family) (Cao et al., 2014)) attributed to some ferns.

On the other hand, investigations of ferns in recent years have put forward the idea of these species as a potential source of bioactive compounds with food interest. In certain Eastern cultures of the European continent fronds of ferns are used as a food source (Langhansova et al., 2021). The young fronds of ferns (named fiddleheads), which generally exhibit a higher total phenol content than the corresponding mature fronds, are a source of nutrients and phytochemicals with a high potential to reduce oxidative stress of diseases associated with ageing (Dvorakova et al., 2021).

**IMAGE 1:**
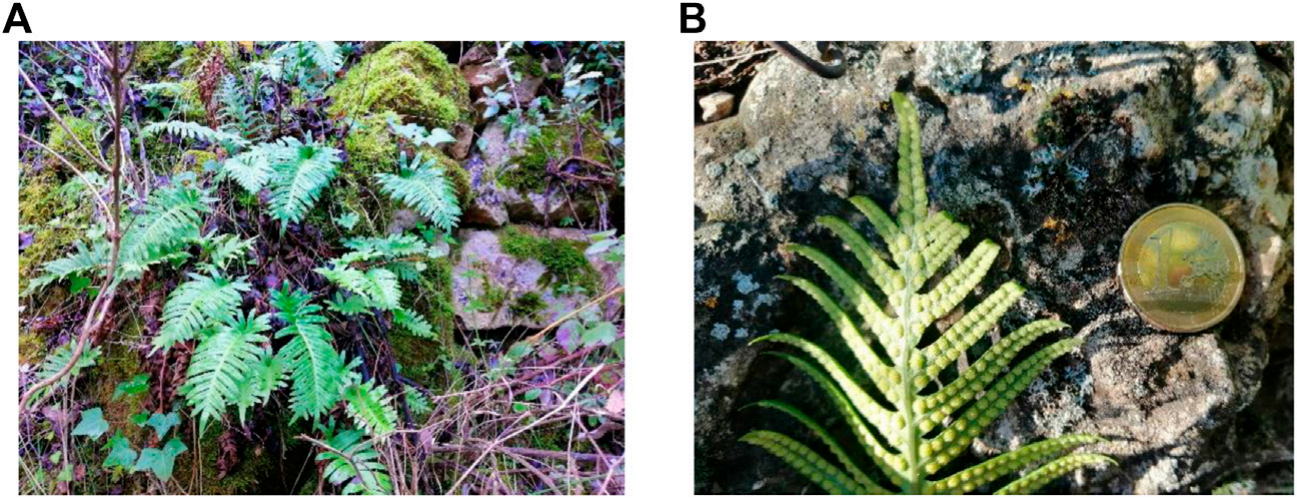
Photography of the face fronds **(A)** and underside frond **(B)** of fresh *Polypodium vulgare* L. (*Polypodiaceae*). Pictures were taken by Adrià Farràs at Prades mountains. The euro coin reflects the dimension of the frond (image 1B).

*Polypodium vulgare* L. (The Plant List, 2021), commonly known as polypody in English or as *polipodio* in Spain for the shape of its fronds as feet (*poly:* many and *podos:* foot), as represented in Image 1, is a fern of the leptosporangiate class belonging to the *Polypodiaceae* family (Bolòs and Vigo, 1984; Khare, 2007; Quer, 2016). *P. vulgare* has been used as medicinal plant in Europe since ancient times. As example, in the middle of the last century the use of *P. vulgare* rhizome infusion as expectorant or diuretic in traditional Polish medicine is reported (Glensk et al., 2019b). Moreover, the fronds of *P. vulgare* have an ethnoveterinary use for treatment variolous, jaundice and parasitic diseases in Spain (Bonet and Vallès, 2007). The use of *P. vulgare* as food is restricted as sweetener in the case of its rhizome (Glensk et al., 2019a). Since 2008, the rhizome of *P. vulgare* has been accepted by European Medicines Agency (EMA) for its use as expectorant herbal medicine in cough and cold and in cases of occasional constipation (EMA, 2008).

In the present study we want to highlight the insufficient number of studies dealing with ferns in comparison with angiosperms to support their potential uses (Chen and Kong, 2005; Cao et al., 2014). Hopefully, these studies will be a turning point for the promotion of traditional and local uses of ferns in Europe and particularly in Spain (Villar and Bonet, 2018).

## Materials and Methods

### Chemicals and Reagents Equations

All reagents were of analytical grade. Trypan blue (0.4%) dye, hydrogen peroxide (H_2_O_2_) 30% w/w, 2,5-diphenyl-3-(4,5-dimethyl-2-thiazolyl) tetrazolium bromide (MTT), dimethylsulfoxide (DMSO), 2,7-dichlorodihydrofluorescein diacetate (DCF) and chlorpromazine hydrochloride (CPZ, CAS No. 69–09-0) were supplied from Sigma-Aldrich (Madrid, Spain). Dulbecco’s modified Eagle’s medium (DMEM) with and without phenol red, fetal bovine serum (FBS), phosphate buffered saline (PBS), l-glutamine solution (200 mM), trypsin-ethylenediaminetetraacetic acid (EDTA) solution (170,000 U/L trypsin and 0.2 g/L EDTA) and penicillin-streptomycin solution (10,000 U/mL penicillin and 10 mg/mL streptomycin) were acquired from Lonza (Verviers, Belgium). All analytical standards used for liquid chromatography analysis shikimic acid, gallic acid, 5-O-caffeoylquinic acid, 3-O-caffeoylquinic acid, (+)-catechin hydrate, (−)-epicatechin, rutin, hyperoside, naringin, quercitrin, 3,5-di-O-caffeoylquinic acid, rosmarinic acid, cinnamic acid, eugenol and *trans*-cinnamaldheyde were purchased from Sigma-Aldrich (Milan, Italy). The 75 cm^2^ culture flasks and 96-well plates were obtained from TPP (Trasadingen, Switzerland). HyClone fetal bovine serum (FBS) was purchased from Thermo Scientific (Northumberland, United Kingdom).

### Plant Material

The fronds of *Polypodium vulgare* L. were collected from the Prades mountains 41°17′34″N 1°02′42″E geographical coordinates (Tarragona, Spain). Previously we verified that this species was reported in the selected area by *Banco de Datos de Biodiversidad de Cataluña* (BDBC, 2015). When the fronds were dried, a sample voucher was stored at Herbarium of Universidad San Jorge (Zaragoza, Spain), *Polypodium vulgare* L.: voucher no. 003-2016.

### Preparation of Methanolic Extract With the Fronds of *Polypodium vulgare* L.

Powdered fronds of the plant material were macerated with methanol for 24 h. After this, the methanolic extract was filtered using a Whatman no. 4 filter paper and to evaporate the solvent, a rotatory evaporator with a thermostatic bath at 30°C was used. This process was repeated three times to obtain the correspondence exhaustion extract as described by ourselves (Farràs et al., 2019). Finally, extracts were conserved at −20°C until we need. Homogenization of the plant extract with the corresponding culture medium was obtained by sonication.

### Phytochemical Characterization by Liquid Chromatography With Diode-Array Detection (HPLC-DAD)

HPLC-DAD studies were performed using a Hewlett-Packard HP-1090 Series II (Palo Alto, CA, United States), equipped with a vacuum degasser, a binary pump, an autosampler and a model 1046A HP photodiode array detector (DAD) following a previous developed method with some modifications (Caprioli et al., 2016). The chromatographic separation was accomplished on a Synergi Polar-RP C18 (4.6 mm × 250 mm, 4 µm) analytical column from Phenomenex (Chesire, United Kingdom). The column was preceded by a security cartridge. The mobile phase for HPLC-DAD (diode array detector) analyses was a mixture of (A) water with 0.1% formic acid (v/v) and (B) acetonitrile with 0.1% formic acid, flowing at 0.8 mL/min in gradient conditions: 0 min, 20% B; 0–15 min, 60% B; 15–20 min, 60% B; 20–25 min, 20% B, 25–30 min, 20% B. The column temperature was set at 30°C and the injection volume was 5 µL. UV spectra were recorded in the range 230–350 nm, where 230 nm was used for quantification of shikimic acid, 256 nm for rutin and hyperoside, 272 nm for gallic acid, 280 nm for (+)-catechin hydrate and (−)-epicatechin, 325 nm for 3-O-caffeoylquinic acid, 5-O-caffeoylquinic acid and 3,5-di-O-caffeoylquinic acid.

Calibration curves of the analysed compounds (Supplementary Material S1) were constructed injecting standard solutions at six different concentrations, i.e., 0.5, 1, 5, 10, 50 and 100 mg/mL, in HPLC-DAD. All the calibration curves of the analysed compounds showed a correlation coefficient greater than 0.9930. The limits of detection and the limits of quantification of the analyzed compounds, expressed in µg/mL were estimated on the basis of 3:1 and 10:1 S/Ns (signal to noise ratio). LODs and LOQs were in the range of 0.03–0.15 and 0.1–0.5 μg/mL, respectively. Retention time stability was utilized to demonstrate the specificity of the method. Reproducibility of the chromatographic retention time for each compound in was examined five times per day over a 5-day period (*n* = 25). The retention times using this method were stable with a percent RSD value of ≤1.33%.

### Cell Culture

The mouse fibroblast cell line, NIH 3T3, and the spontaneously immortalized human keratinocyte cell line, HaCaT, were used in all *in vitro* experiments. In the case of cytotoxic assay, cell viability also was evaluated by the cervical cancer cell line HeLa, the liver cancer cell line HepG2, the breast cancer cell line MCF-7 and lung cancer cell line A549. 3T3 and A549 were purchased from Sigma-Aldrich as a worldwide provider of European Collection of Authenticated Cell Cultures (ECACC), whereas HaCaT, HeLa and MCF-7 were obtained from Eucellbank (Celltec-Universitat de Barcelona, Spain). HepG2 cell line was kindly donated by Dr. Borràs of Experimental Toxicology and Ecotoxicology Platform (UTOX-CERETOX) of Parc Científic of Universitat de Barcelona.

Cell maintenance and culture were performed in Dulbecco’s Modified Eagle’s medium (DMEM) supplemented with 10% heat-inactivated fetal bovine serum (FBS), 2 mM l-glutamine and 100 U/mL:100 U/mL streptomycin-penicillin mixture (10% FBS-DMEM) at 37°C in a 5% carbon dioxide (CO_2_)-humidified incubator. Cells were routinely subcultured in 75 cm^2^ flasks.

Experimental treatments were performed when cells reached 80% of confluence, culture medium was removed, cells were rinsed with PBS and then detached by trypsinization (trypsin-EDTA). From the cellular suspension obtained and after adjusting cell density at 1 x 10^5^ cells/mL, 100 were seeded in 96 well microplates and incubated overnight (37°C and 5% CO_2_). Cell density was adjusted by counting the number of viable cells with the trypan blue (0.4%) dye exclusion.

### Cytotoxicity Activity of Methanolic Extract of *Polypodium vulgare* L. in Non-Tumoral and Tumoral Cells Lines

Non-tumoral (3T3 and HaCaT) and tumoral cells lines (HeLa, HepG2, MCF-7 and A549) were treated for 24 h (37°C and 5% CO_2_) with increasing concentrations of methanolic extract 0.01, 0.1, 1 and 2 mg/mL *P. vulgare* in 5% FBS-DMEM. For each independent experiment and plate, untreated cells (maintained with culture medium) were included as negative controls. Cytotoxicity of PVM was determined by the NRU and MTT assays.

#### Determination of Cell Viability by Neutral Red Uptake and 2,5-Diphenyl-3-(4,5-Dimethyl-2-Thiazolyl) Tetrazolium Bromide Assays

Cell viability were determined by the NRU and MTT methods after treatments.

The Borenfreund and Puerner protocol for the determination of cell viability by NRU has been followed with some described adaptations (Borenfreund and Puerner, 1985). Once the incubation time of the cells with the treatments had elapsed, the supernatant was extracted from each well and 100 μl of NR solution was applied (0.05 mg/mL in serum-free DMEM without phenol red). After 3 hours, the supernatant was removed by inversion from the plate and 100 µl of the developer NR solution was added. In the developer solution, the formaldehyde was replaced by an acidic ethanol solution (Riddell et al., 1986). The quantification of the remnant NR, which corresponds to the NRU bound to the lysosomes, is proportional to the viable cells (Weyermann et al., 2005). After 5–10 min of shaking the plate, the absorbance was obtained at 550 nm, by means of the Tecan Sunrise microplate reader (Männedorf, Switzerland).

The MTT assay based on the experimental protocol of Mosmann (Mosmann, 1983) was used with the some previous adaptations (Zanette et al., 2011); 100 µL of an MTT solution (0.5 mg/mL in serum-free DMEM without phenol red) was added in each well following incubation of the plates for at least 3 h in cell culture incubation conditions (37°C and 5% CO_2_). At the end of incubation, supernatant was removed and 100 µL of the organic dissolvent dimethyl sulfoxide (DMSO) was added to each well to dissolve the formazan crystals (Präbst, 2017). The amount of soluble formazan is proportional to the number of cells with optimal mitochondrial activity (Fotakis and Timbrell, 2006). Absorbance was measured at 550 nm using a Tecan Sunrise microplate reader (Männedorf, Switzerland), previous homogenization of the well content by gently shaking each microplate during 5 min at 100 rpm/min.

*Cell viability* for NRU and MTT assays were calculated using the following equation:

Cell viability (%)=(Acontrol−AsampleAcontrol) x 100

where *A*_*control*_ and *A*_*sample*_ are the absorbance of the control and each sample, respectively.

### Cytoprotective Activity in 3T3 and HaCaT Cell Lines

Potential protective effect of the extract was then explored in the non-tumoral cell lines against oxidative stress induced by hydrogen peroxide (Cásedas et al., 2020). Cells were pre-treated with 0.01, 0.1, 1 and 2 mg/mL PVM (100 μL) dissolved by 5% FBS-DMEM for 24 h following addition of H_2_O_2_ (in 5% FBS-DMEM) at a final concentration 2 mM for 2.5 h. Finally, cell viability was determined by NRU and MTT assay. In each microplate negative and positive controls were included. In this case, positive controls consist of cells treated by H_2_O_2_ at 2 mM during 2.5 h without previous pre-treatment with the extracts.

*Cytoprotective activity* was calculated as follows:

Cytoprotective activity (%)=(CVPVM−H2O2−CVH2O2CVPVM−H2O2) x 100

where *CV* is the cell viability for each condition described in the formula.

#### Cellular Repair Activity in 3T3 Cells

Cellular repair properties were evaluated using 100 μL H_2_O_2_ at 2 mM during 2.5 h before applying PVM at different concentrations (0.01, 0.1, 1 and 2 mg/mL). Cell viability was assessed by NRU and MTT assays 24 h after incubation with the treatments.

*Cellular repair activity* was calculated as follows:

Cellular repair activity (%)=(CVPVM−H2O2−CVH2O2CVPVM−H2O2) x 100

where *CV* is the cell viability for each condition described in the formula.

### Phototoxicity Activity of Methanolic Extract of *Polypodium vulgare* L. in 3T3 and HaCaT Cell Lines

In parallel with the study of the potential cytoprotective protection of the extract, we have explored the potential phototoxic activity of PVM. For this purpose we followed the Organization for Economic Cooperation and Development (OECD) TG 432 (2019) (OECD, 2019) with some adaptions.

Briefly, 3T3 and HaCaT cells were plated at a density of 1 x 10^5^ cells/mL (100 μL) in a 96 well microplate in 10% FBS-DMEM for 24 h. Cells were treated with PVM samples and incubated for 1 h (37°C, 5% CO_2_) before being irradiated with 1.8 J/cm^2^ of ultraviolet A (UVA) light. To avoid as much as possible protein interferences with protein and light absorbing components, PVM samples were solubilized in serum-free DMEM without phenol red. Moreover, for comparative purposes and to correctly interpret the data, negative controls consisted in non-treated cells whereas positive controls consisted in cells treated with the well-known phototoxic chemical chloropromazine.

After irradiation, cell media was replaced for 100 μL of fresh medium (10% FBS-DMEM) and cell viability was determined after 24 h of incubation by the NRU and MTT colorimetric assays.

Light exposure was performed in a photostability UV chamber (58 × 34 × 28 cm) equipped with three UVA lamps Actinic BL TL/TL-D/T5 (Philips, 43 V, 352 nm, 15 W) as described by our research group (Martínez et al., 2013). Dosage and time exposition of cells to UVA light was regularly settled thanks to a photoradiometer Delta OHM provided with a UVA probe (HD2302 - Italy). We followed the equation:

E (Jcm2)=t(s) x P (Wcm2)

where *E* stands for ultraviolet dose, *t* represents the time expressed in seconds and, finally, *P* is the lamp potency.

### Intracellular Reactive Oxygen Species (ROS) Induced by H_2_O_2_ of Methanolic Extract of *Polypodium vulgare* L. in 3T3 and HaCaT Cell Lines

ROS production was tested accordingly (Ferreira et al., 2018). After the incubation of the cells with the different concentrations of the extract for 24 h as reported in previous sections for *in vitro* assays, cells were washed twice with PBS and DCF (100 µM) was applied to each well for 45 min (37°C and 5% CO_2_). DCF that has not penetrated cells was removed by washing twice with cell culture medium and then H_2_O_2_ (1 and 2 mM) was added to induce oxidative stress. The fluorescence intensity of the oxidized product of DCF was registered (λ_excitation_ 480 nm; λ_emision_ 530 nm) at 0, 1, 2 and 3 h by a plate reader ThermoFisher SCIENTIFIC VARIOSKAN LUX (ThermoFisher SCIENTIFIC, Waltham, Massachusetts, United States). Results were expressed as *Fluorescence Intensity (FI)* which have adimensional units. The *FI*_*z h Vs 0 h*_ were calculated as follows:

Fluorescence Intensityz h Vs 0 h (FIz h Vs 0 h)=(FIz h−FI0 hFIz h) x 100

where *FI*_*z h*_ is the intensity fluorescence at *z* h (*z* as 1, 2 or 3 h) of incubation and *FI*_*0 h*_ the amount fluorescence intensity at 0 h.

The *FI* for each specific time was calculated using this formula:

FI=Fluorence480 nm (excitation)Fluorence530 nm (emision)

The *∆ROS*, which have adimensional units for FI, was obtained using the following formula:

$$\Delta \mathrm{RO}S_{H2O2}=\Delta \mathrm{RO}S_{\mathrm{PVM with DCF}-H2O2}-\Delta \mathrm{RO}S_{\mathrm{DCF}-H2O2}$$

### Statistical Analysis

All experiments were carried out in triplicates and almost three independent experiments were assayed, on different days, except for the cytoprotection PVM HaCaT against 2 mM H_2_O_2_ (2.5 h) MTT for which the results correspond to *n* = 2 experiments. Statistical significance for MTT cell viability and fluorescence intensity was analysed by using GraphPad Prism version 7, San Diego, CA, United States. Data are presented as mean ± standard error. Activities have been compared using a two-way analysis of variance (ANOVA) by Bonferroni. Statistical differences were considered as follows: *p* ≤ 0.05 (*), *p* ≤ 0.01 (**), *p* ≤ 0.001 (***) and *p* ≤ 0.0001 (****).

## Results

### Phytochemical Characterization by Liquid Chromatography With Diode-Array Detection (HPLC-DAD)

Different types of polyphenols were monitored in the extract. The extract proved to contain different types of phenolic acids and flavonoids, as observed in Table 1 (77,823.7 mg/kg). However, naringin, quercitrin, rosmarinic acid, cinnamic acid, eugenol and *trans*-cinnamaldheyde were not detected.

**TABLE 1:** Quantitative determination of metabolites in the methanolic extract *Polypodium vulgare* L. by HPLC-DAD reported at 272 nm.

No.	Phytochemicals	Quantity (mg/kg extract)^a^ ^,^ ^b^
1	Shikimic acid	5,339.3 ± 70.6
2	Gallic acid	1791.3 ± 38.3
3	5-O-caffeoylquinic acid	256.5 ± 12.1
4	3-O-caffeoylquinic acid	58,778.3 ± 417.7
5	(+)-Catechin hydrate	3,879.8 ± 153.3
6	(−)-Epicatechin	7,158.5 ± 88.8
7	Rutin	422.7 ± 30.4
8	Hyperoside	91.3 ± 11.7
9	3,5-di-O-caffeoylquinic acid	106.0 ± 15.5
	Total content	77,823.7 ± 838.4

a Results are expressed in mg/kg dry extract, n = 3.

b Cinnamic acid, eugenol, naringin, quercitrin, rosmarinic acid and trans-cinnamaldehyde were not detected.

**FIGURE 1:**
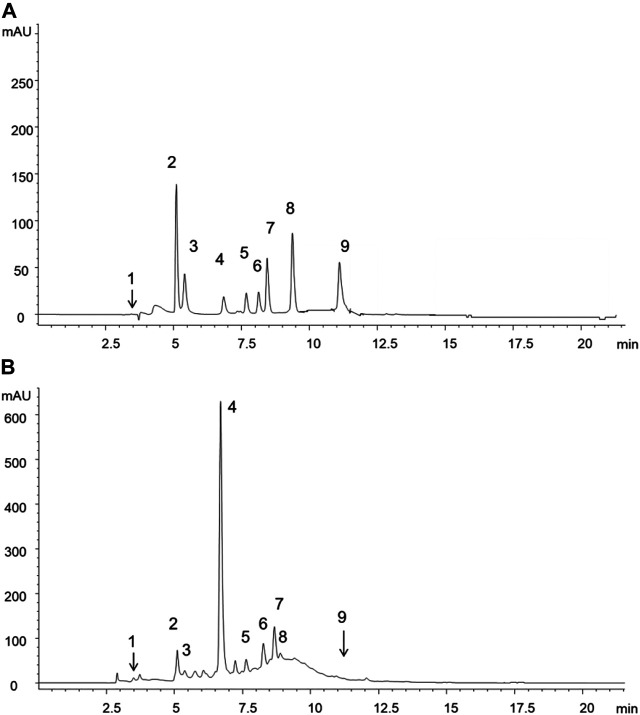
HPLC-DAD chromatograms reported only at 272 nm for sake of clarity and corresponding to **(A)** standard mixture solution **(B)** extract of methanolic fronds extract of *Polypodium vulgare* L. List of compounds: 1 = shikimic acid, 2 = gallic acid, 3 = 5-O-caffeoylquinic acid, 4 = 3-O-caffeoylquinic acid, 5 = catechin, 6 = epicatechin, 7 = rutin, 8 = hyperoside, 9 = 3,5-di-O-caffeoylquinic acid.

The major constituents in the extract, as seen in Figure 1, were 3-O-caffeoylquinic acid (58,778.3 mg/kg), epicatechin (7,158.5 mg/kg), shikimic acid (5,339.3 mg/kg) and catechin (3,879.8 mg/kg) which were phenol acids. The peculiar secondary metabolites found were hyperoside and 3,5-di-O-caffeoylquinic acid, with low concentrations (91.3 and 106.0 mg/kg respectively).

### Cytotoxic Activity in Non-Tumoral and Tumoral Cell Lines

A set of cytotoxic assays was carried out to determine the cytotoxic potential; however, data on NRU method were not shown.

**FIGURE 2:**
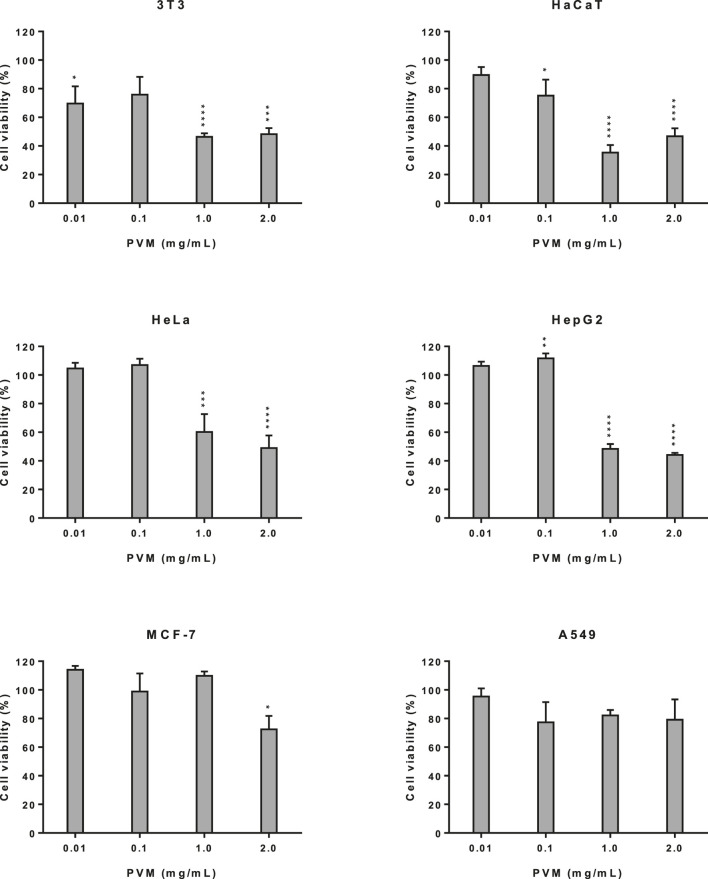
Cytotoxicity activity of PVM in 3T3, HaCaT, HeLa, HepG2, MCF-7 and A549 cell lines by MTT assay and expressed as percentage of cell viability respect to control cells. Results are expressed as mean ± standard error of *n* = 3. Control cells were maintained only with culture medium. A two-way analysis of variance (ANOVA) and a Bonferroni *post hoc* assay have been performed. Statistical differences were considered as follows: *p ≤ 0.05, **p ≤ 0.01, ***p ≤ 0.001 and ****p ≤ 0.0001 compared with no treated cells (negative control).

Figure 2 shows cell viability obtained by the MTT assay for the different cell lines described here. First, we evaluated the cytotoxic activity of the PVM in 3T3 and HaCaT as a representation of non-tumoral cell lines. In both cell lines a marked increase in cytotoxicity was observed, at concentrations of 1 and 2 mg/mL PVM compared to 0.01 and 0.1 mg/mL PVM but with a slightly higher cytotoxicity activity of PVM in HaCaT (35.3%) than in 3T3 (46.4%) at 1 mg/mL PVM.

The cytotoxicity study was extended to tumoral cells such us HeLa, HepG2, MCF-7 and A549 cells. As we can observe in Figure 2, PVM on HeLa and HepG2 cells presents a similar cytotoxic behaviour, presenting a significant decrease in cell viability at the highest concentration assessed, being this decrease slightly higher in HepG2 (44.2%) than HeLa (49.0%). No cytotoxic effects have been determined at 0.01 and 0.1 mg/mL of the extract.

For MCF-7 and A549 cells (Figure 2) no statistical differences among cell viability is observed at the different concentrations studied of PVM, although values show a slight decrease to 72.5% in MCF-7 at 2 mg/mL and 77.3% in A549 at 0.1 mg/mL.

The present results exhibited that, cytotoxicity effects only appear at 1 and 2 mg/mL PVM in 3T3, HaCaT, HeLa and HepG2.

### Cytoprotective Activity in 3T3 and HaCaT Cell Lines

Before the potential cytoprotective activity were studied, the deleterious effects of H_2_O_2_ in the cells were initially determined. From this previous assay (data not shown), we have established that cell viability obtained at 2 mM of H_2_O_2_ for 2.5 h (30.5 and 41.0% for 3T3 and HaCaT respectively) allows us to evaluate potential beneficial effects of PVM.

**FIGURE 3:**
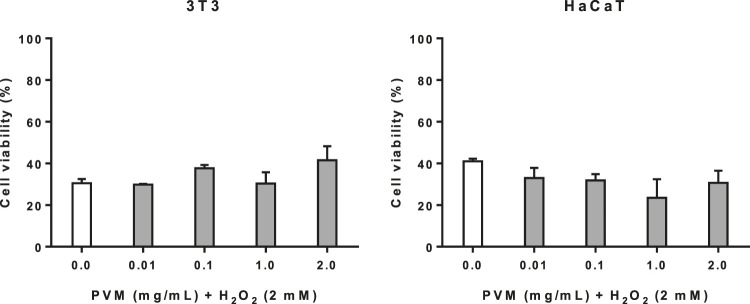
Cytoprotective activity of PVM in 3T3 and HaCaT cell lines for 2 mM H_2_O_2_ during 2.5 h by MTT assay and expressed as percentage of cell viability respect to untreated cells control. H_2_O_2_ cell viability was used as positive control. Results are expressed as mean ± standard error of *n* = 3 and *n* = 2 respectively. A two-way analysis of variance (ANOVA) and a Bonferroni *post hoc* assay have been performed. No statistically significant differences were found.

As observed in Figure 3, cell viability in 3T3 increases mainly in parallel to PVM concentrations indicating some cytoprotective effect although not statistically significant, being this cytoprotective activity of 18.9 and 26.5% at 0.1 and 2 mg/mL, respectively. In the case of HaCaT, no cytoprotective effect has been observed in any of the concentrations tested.

#### Cellular Repair Activity in 3T3 Cells

**FIGURE 4:**
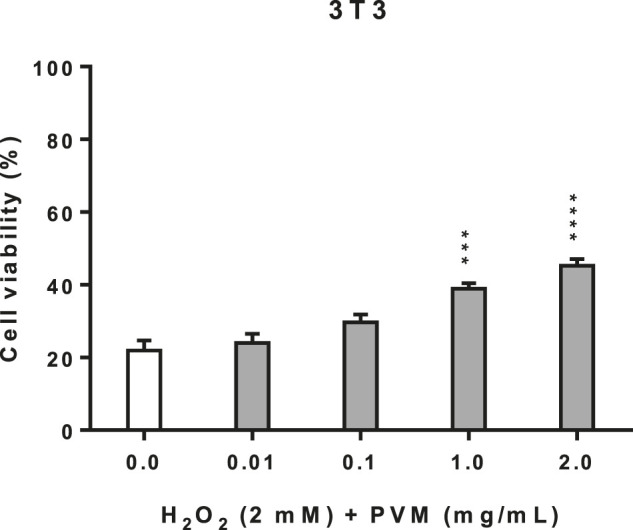
Cellular repair activity of PVM in 3T3 cell line for 2 mM H_2_O_2_ during 2.5 h by MTT assay and expressed as percentage of cell viability respect to untreated cells control. H_2_O_2_ cell viability was used as positive control. Results are expressed as mean ± standard error of *n* = 3. A two-way analysis of variance (ANOVA) and a Bonferroni *post hoc* assay have been performed. Statistical differences were considered as follows: ***p ≤ 0.001 and ****p ≤ 0.0001 compared with positive control.

In the cellular repair assay, we have used the same conditions of H_2_O_2_ as in cytoprotection assay (2 mM H_2_O_2_ for 2.5 h). As we can observe in Figure 4, there is an increase in cell viability as PVM concentration rises; however, this discrete reparation effect is proportional to the concentration of the extract.

### Phototoxicity Activity of Methanolic Extract of *Polypodium vulgare* L. in 3T3 and HaCaT Cell Lines

**FIGURE 5:**
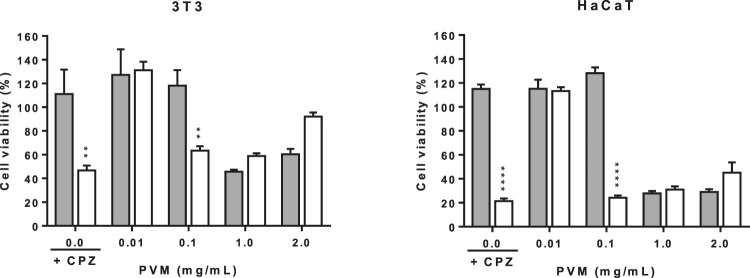
Phototoxicity activity of PVM in 3T3 and HaCaT cell lines by MTT assay and expressed as percentage of cell viability respect to the correspondent control cells. Chloropromazine cell viability was used as positive control. Gray columns correspond to cells non exposed to UVA light and white columns correspond to cells exposed to 1.8 J/cm^2^ of UVA light. Results are expressed as mean ± standard error of *n* = 3. A two-way analysis of variance (ANOVA) and a Bonferroni *post hoc* assay have been performed. Statistical differences were considered as follows: **p ≤ 0.01 and ****p ≤ 0.0001 compared with correspondence no irradiated/irradiated positive control.

The validity of the assay has been determined by calculating the ratio of cell viability in irradiated respect to non-irradiated conditions of both negative and positive control cells. Doses of 1.8 J/cm^2^ of UVA light affects the viability of non-treated cells that decrease in both cell lines. In the phototoxicity assay is important to consider that cell viability of control cells not treated but irradiated present a cell viability of about 63.3 and 75.0% respect from the non-irradiated ones for 3T3 and HaCaT respectively. Indicating that 3T3 are much sensitive to light than HaCaT and that interpretation of data should be interpreted carefully, as seen in Figure 5. However, the effect of the photosensitizer CPZ in 3T3 and HaCaT is confirmed by the important drop of cell viability when exposed to UVA respect not exposed to UVA in both cell lines, 26.9 and 13.7% cell viability respectively. Considering these ratios, the viability obtained when cells were exposed to UVA in the presence of PVM is considered.

In general, PVM did not show phototoxic behaviour in the assayed conditions, except at 0.1 mg/mL. At this concentration, there is a decrease in viability when cells are exposed to light.

### Intracellular Reactive Oxygen Species (ROS) Induced by H_2_O_2_ of Methanolic Extract of *Polypodium vulgare* L. in 3T3 and HaCaT Cell Lines

**FIGURE 6:**
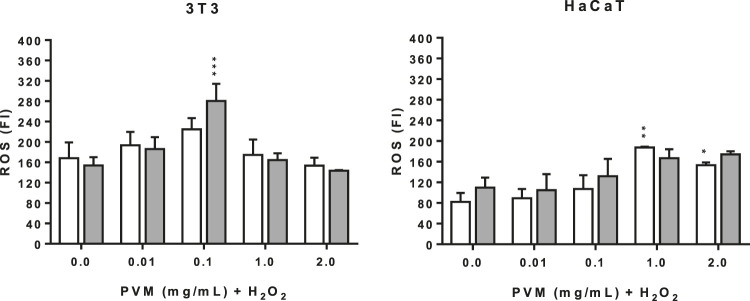
Intracellular ROS induced by 1 and 2 mM H_2_O_2_ for 2 h treatment with PVM in 3T3 and HaCaT cells. H_2_O_2_: positive control. White columns correspond to 1 mM H_2_O_2_ and gray columns correspond to 2 mM H_2_O_2_. Results are expressed as mean ± standard error of *n* = 3. A two-way analysis of variance (ANOVA) and a Bonferroni *post hoc* assay have been performed. Statistical differences were considered as follows: *p ≤ 0.05, **p ≤ 0.01 and ***p ≤ 0.001 compared with the correspondent positive control.

The production of ROS was explored by the fluorescence intensity with the DCF probe. As shown in Figure 6, for each cell line was obtained the same tendency of ROS production at 2 h, with similar pattern recorded at 1 and 3 h (data not shown).

The production of ROS in the conditions tested here are significantly different for both cell lines. In the case of 3T3, positive controls show similar values of ROS production at the two concentrations of H_2_O_2_ but higher than those obtained in the case of HaCaT. This observation suggests that keratinocytes are less sensitive than 3T3.

In the case of 3T3 there is a peak of intracellular ROS production in the presence of PVM at 0.1 mg/mL, which is more pronounced in cells treated with 2 mM H_2_O_2_, followed by an important reduction at 1 mg/mL and, finally, reverted at 2 mg/mL. This pattern is independent of the final concentration of H_2_O_2_. In contrast, the production of ROS is dose dependent in the case of HaCaT, except at 2 mg/mL PVM at 1 mM H_2_O_2_. One explanation to this different behavior between the two cell lines can be attributed to the presence of different protective antioxidant systems and that can also explain the ROS production showed by the positive controls.

## Discussion

Despite the significant biological activities attributed to the *Polypodiaceae* family reported in various studies, such as antidiabetic (*Phymatopteris triloba* (Houtt.) Pic. Serm.) (Chai et al., 2013), anticancer (*Polypodium leucotomos*) (Gonzalez et al., 2010) and anti-inflammatory activities (*Polypodium leucotomos*) (Choudhry et al., 2014), there are many other *Polypodiaceae* ferns not yet characterized or studied specifically for their potential food or medical uses. This is the case of *Polypodium vulgare* L. Probably, the most studied *Polypodiaceae* fern is *Polypodium leucotomos* because of the commercialization of its standardized aqueous extract fronds (PLE) (known as Fernblock and formulated in cosmetic products and food supplements used to protect from sun exposition) (Palomino, 2015) and its standardized ethanolic dry extract rhizome (known as the oral medicine Difur for skin inflammatory disorders) (AEMPS, 2013). In addition, the aqueous extract fronds of *P. leucotomos* has been reported as a natural product for the treatment of skin alterations such as photodermatosis, adjunctive treatment of melasma (as chemopreventive), vitiligo, psoriasis vulgaris and atopic dermatitis, among others (Choudhry et al., 2014; Parrado et al., 2018; Thompson and Kim, 2020).

As stated by Messeguer (Messeguer et al., 1998), the two major drugs used of *P. vulgare* are rhizomes and fronds as reported for other species of *Polypodium* (Liu et al., 1998). In the present study we have obtained a methanol extract from the fronds of the fern. Some studies reported the isolated phytochemical composition of the rhizome, describing the different types of phytochemicals namely flavonoids as flavan-3-ol derivatives (Glensk et al., 2019a), triterpenoids hydrocarbons, triterpenoids alcohols of the cycloartane group, saponin glycosides (Arai et al., 1989; Arai et al., 1991), phytoecdysteroids (Messeguer et al., 1998) and others (Dar et al., 2012). However, to date no published work or study has dealt with the composition of the fronds of *P. vulgare* except for the one of Messeguer describing the presence of some phytoecdysteroids (Messeguer et al., 1998). Among the few articles reporting the composition and bioactivity of this fern, Sofiane et al. (2015) describes antioxidant, antimicrobial and anti-inflammatory activities attributing these activities to different groups of phytochemicals but unspecifying the part of the plant used (drug plant). Also, Glensk et al. (2019b) reports the antimicrobial activity of the rhizome attributed to osladin. It is widely known that the production of secondary metabolites is a response to environmental conditions (environmental stress, period of the year, among other variables) to which the plant is exposed (Wu et al., 2017). To eliminate this variable, the fronds from which the extract was obtained in the present study were collected at the same time of the year (November 2016). Using HPLC-DAD, we have determined a high number of phenolic related compounds and a small fraction of flavonoids (less than 15% of the total polyphenolic species) represented by (+)-catechin hydrate, (−)-epicatechin, rutin and hyperoside. This observation agrees with our previous study using thin layer chromatography (TLC) (Farràs et al., 2019). It is well known that flavonoids, due to their radical scavenging ability provided by its chemical structure described elsewhere (Rice-Evans et al., 1996; Prochazkova et al., 2011; Wen et al., 2014), have a greater antioxidant capacity than certain phenolic acids (Leopoldini et al., 2011); however, flavonoids are residual components of our extract. Other phenolics such as *p*-coumaric, ferulic, caffeic, vanillic and chlorogenic acids, were reported as the major polyphenol phytochemicals in *P. leucotomos* (Gombau et al., 2006; García et al., 2006). Another important aspect to consider is the synergy that the different phytochemicals present in an extract against the oxidative damage (Yen et al., 2013; Naik and Sellappan, 2020).

In the Asian continent, a variety of ferns have been used as remedies as the case of the Gusuibu ferns (Chang et al., 2007). Nevertheless, other fern species, such as *Pteridium aquilinum* (L.) Kuhn (*Dennstaedtiaceae* family), contain ptaquiloside, a toxic compound that can cause cancer (da Costa et al., 2012; O’Connor et al., 2019). For this reason, the objective of the present work is to study the bioactivity of the polar constituents of *P. vulgare* including its cytotoxic potential in non-tumoral (3T3 and HaCaT) and tumoral cells (HeLa, HepG2, MCF-7 and A549). The protective activity of the extract against oxidative stress is also studied by different assays. Four concentrations of the extract (0.01, 0.1, 1 and 2 mg/mL) were selected in the present study considering physiological and non physiological concentrations for a better understanding.

There are many viability assays used to evaluate cytotoxic activity of different substances and products. NRU has been proven to be a sensitive assay to study the cytotoxic activity and potential protection of procyanidin fractions from grape and pine against the H_2_O_2_ insult (Ugartondo et al., 2007; Mitjans et al., 2011). In addition, MTT is regarded as a gold standard of cytotoxicity assays as it is highly sensitive and a high-throughput screening assay. However, recently Karakas have described an interfering effect of the methanolic extract of different Turkish’s plant extracts resulting in false-positive viability (Karakas et al., 2017). In this sense, potential interferences of our fern extracts with the MTT assay were performed previously to study their cytotoxic activity. Taken together these aspects, we considered studying the biological activity of our extracts by these two assays. In our case, NRU failed to be sensitive according to our MTT data. However, our results can indicate that mechanism of cytotoxic behaviour of PVM does not include lysosomal damage.

The potential use of ferns to prevent or treat tumoral processes, as the case of some Asiatic fern species, has been demonstrated by the cytotoxic, pro-apoptotic or cell cycle-arresting effects of non-characterized plant extracts (Tomsik, 2014). In our case, no relevant cytotoxic effects have been reported for the extract in the different cell lines, except in the case of the HaCaT cells, but only at the very high concentrations. The phytochemical characterization by HPLC-DAD indicates that epicatechin is the second major compound of the flavonoid components of the extract. Moreover (Cao et al., 2014), a strong cytotoxic behaviour of an ethanol extract of the whole fern *Davallia cylindrica* Ching in A549 cells has been described and attributed to the high content of quercitrin and some of its derivatives. Using HPLC-DAD we failed to detect quercitrin (a glycosylated derivative of quercetin), which can explain the absence of relevant cytotoxicity (Farràs et al., 2019). Results obtained with 3T3 and HaCaT cell lines open the possibility to validate the traditional use of this species in the Sobrarbe region as disinfectant and wound healing (Huesca) (Villar and Bonet, 2018), mostly considering that fibroblasts, the most common cells in connective tissue, play a critical role in wound healing and keratinocytes form epidermis, which is a biological and physical barrier against injuries. These results are in line with the fact that the pteridophytes presents an antibiotic properties (Banerjee and Sen, 1980).

The protective effect of the extract was assessed in this study against hydrogen peroxide in 3T3 and HaCaT cells. Our results show that H_2_O_2_ causes slightly higher mortality in 3T3 (30.5% cell viability) than in HaCaT (41.0% cell viability), which can be explained by a higher antioxidant defence system on keratinocytes than fibroblasts (Pérez et al., 1995). This minor mortality in HaCaT can justify that the extract failed to present cytoprotective capacity in such cell line. We have observed a discrete cytoprotective effect of PVM in the 3T3 cells being the first report that deals with this kind of assays using a fern extract from the *Polypodiaceae* family. However, Gomes et al. (2001) and Gombau et al. (2006) have described the potential antioxidant activity of *P. leucotomos*, by different *in vitro* methods.

H_2_O_2_ is recognized as a pleiotropic compound in the induction of oxidative stress (Sies and Jones, 2020). However, its effect on the induction of oxidative stress differs in whether a pre-treatment (cytoprotective activity), co-treatment or post-treatment (cellular repair activity) trial is performed (Siddiqui et al., 2011). In the post-treatment test, as result of an oxidative stress that triggers severe cellular damage, cells are sometimes unable to regain redox homeostasis despite being subsequently treated with an antioxidant agent. Considering our results of cytoprotection, the cellular repair test was only performed in the 3T3 cell line. This assay showed that there is a significant increase in cellular viability directly proportional to PVM concentration suggesting the capacity of the extract to induce cellular repair mechanisms.

Other authors have reported that *P. leucotomos* was able to protect human fibroblast from cytoskeletal disarrangements induced by UVA light (1 J/cm^2^) (Alonso-Lebrero et al., 2003). Moreover, Philips et al. (2003) reported that a concentration lower than 0.1% improves cellular membrane integrity and inhibits MMP-1 on fibroblast and keratinocytes thus suggesting its potential use in prevention on skin photoaging. However, before studying the potential photoprotective activity of the extract, we should discard any phototoxic reactions. In the present study, the determination of phototoxicity is based on the OECD TG 432 (OECD, 2019), where the BALB/c 3T3 cell line has been replaced by NIH 3T3 and we included the HaCaT cell line and the determination of cell viability by MTT as previously reported (Baccarin et al., 2015). In general, we can conclude that PVM is not phototoxic although the decrease in cell viability at 0.1 mg/mL in both cell lines needs to be clarified. One interpretation could arise from the direct toxic effects of UVA light over the cells that can be reverted by the presence of the extract at high concentrations but not at moderate ones as 0.1 mg/mL PVM. Further investigation should be conducted to explore the cellular mechanisms that are activated. Contact time of the extract also should be considered, thus in this phototoxicity test is 1 h plus the time of UVA exposition, whereas in the rest of assays the extract remains approximately 24 h in contact with cells.

It is known that UV damages mitochondrial DNA (Hseu et al., 2015), for this reason it would be interesting to also evaluate the potential phototoxic or photoprotective activity of the PVM by other assays such as the comet assay in a similar way as previously described for pomegranate seed oil nanoemulsion in HaCaT (Baccarin et al., 2015). Currently, the mechanisms by which *P. leucotomos* protects against UV-induced DNA damage, such as overexpression of the p53 gene, have already been described (Parrado et al., 2020).

Hydrogen peroxide is an oxidative agent that promote the endogenous generation of ROS in diverse cell lines (Uguz et al., 2016). If high ROS concentrations trigger cell death, the loss of mitochondrial functionality begins with the consequent apoptosis (Moloney and Cotter, 2018). In our case, there is an increase of ROS production in both cell lines except at 2 mg/mL. This increase of ROS production observed here and, particularly in HaCaT cells apart from the lowest concentration extract tested (0.01 mg/mL PVM), may be explained by the pro-oxidant effect of polyphenols (Santos et al., 2018). The mechanism why this ROS can diminish cell viability needs to be clarified and further explored in other tumoral cell lines as a first step to better characterize the chemotherapeutic potential of PVM (Skibola and Smith, 2000).

Additionally, in recent years, phenolic compounds are positioning themselves as the reference antioxidant substances of natural origin (Virgili and Marino, 2008; Piccolella et al., 2019). Thus, certain foods and plant extracts with high antioxidant properties are positioned as a powerful adjuvant treatment to counteract the adverse drug reactions (ADR) associated with the chemotherapy (Oyenihi and Smith, 2019).

## Conclusion

The absence of cytotoxicity at physiological concentrations determined in the six cell lines of the present study, together with the high concentration of phenolics in the fronds of *P. vulgare*, is decisive to confirm this fern as a source of bioactive and antioxidant compounds with pharmaceutical applications. Some traditional uses of *P. vulgare*, such as the wound healing benefits, have still not been proved but this is the first time that the fronds are positioned as potential bioactive agents. This article could also be an inflexion point to justify further research of the fronds of *P. vulgare*.

## Data Availability

The raw data supporting the conclusions of this article will be made available by the authors, without undue reservation.

## Glossary

3T3 = NIH 3T3: mouse fibroblast cell line
A549: lung cancer cell line
ADR: Adverse Drug Reactions
CO_2_: carbon dioxide
CV: Cell Viability
CPZ: chlorpromazine hydrochloride
E: ultraviolet dose
EMA: European Medicines Agency
DCF: 2,7-dichlorodihydrofluorescein diacetate
DMEM: Dulbecco’s Modified Eagle’s Medium
DMSO: dimethyl sulfoxide
EDTA: ethylenediaminetetraacetic acid
ECACC: European Collection of Authenticated Cell Cultures
FBS: Fetal Bovine Serum
FI: Fluorescence Intensity
HaCaT: spontaneously immortalized human keratinocyte cell line
HeLa: cervical cancer cell line
HepG2: liver cancer cell line
HPLC-DAD: liquid chromatography with diode-array detection
H_2_O_2_: hydrogen peroxide
MCF-7: breast cancer cell line
MTT: 2,5-diphenyl-3-(4,5-dimethyl-2-thiazolyl) tetrazolium bromide
NR: Neutral Red
NRU: Neutral Red Uptake
OECD: Organisation for Economic Cooperation and Development
PBS: Phosphate Buffered Saline
PL: *Polypodium leucotomos*
PLE: aqueous extract of *Polypodium leucotomos*
PVM: methanolic extract of *Polypodium vulgare* L. fronds
ROS: Reactive Oxygen Species
RNS: Reactive Nitrogen Species
TLC: Thin Layer Chromatography
UV: ultraviolet
UVA: ultraviolet A
