# Supplementary material for: Polypodium vulgare L. (Polypodiaceae) as a Source of Bioactive Compounds: Polyphenolic Profile, Cytotoxicity and Cytoprotective Properties in Different Cell Lines
Source: Front Pharmacol. 2021 Sep 16;12:727528. doi: 10.3389/fphar.2021.727528 (PMC8482143; doi:10.3389/fphar.2021.727528)
Supplement: Supplementary file 1 [file Presentation1.PPTX]

## Slide 1
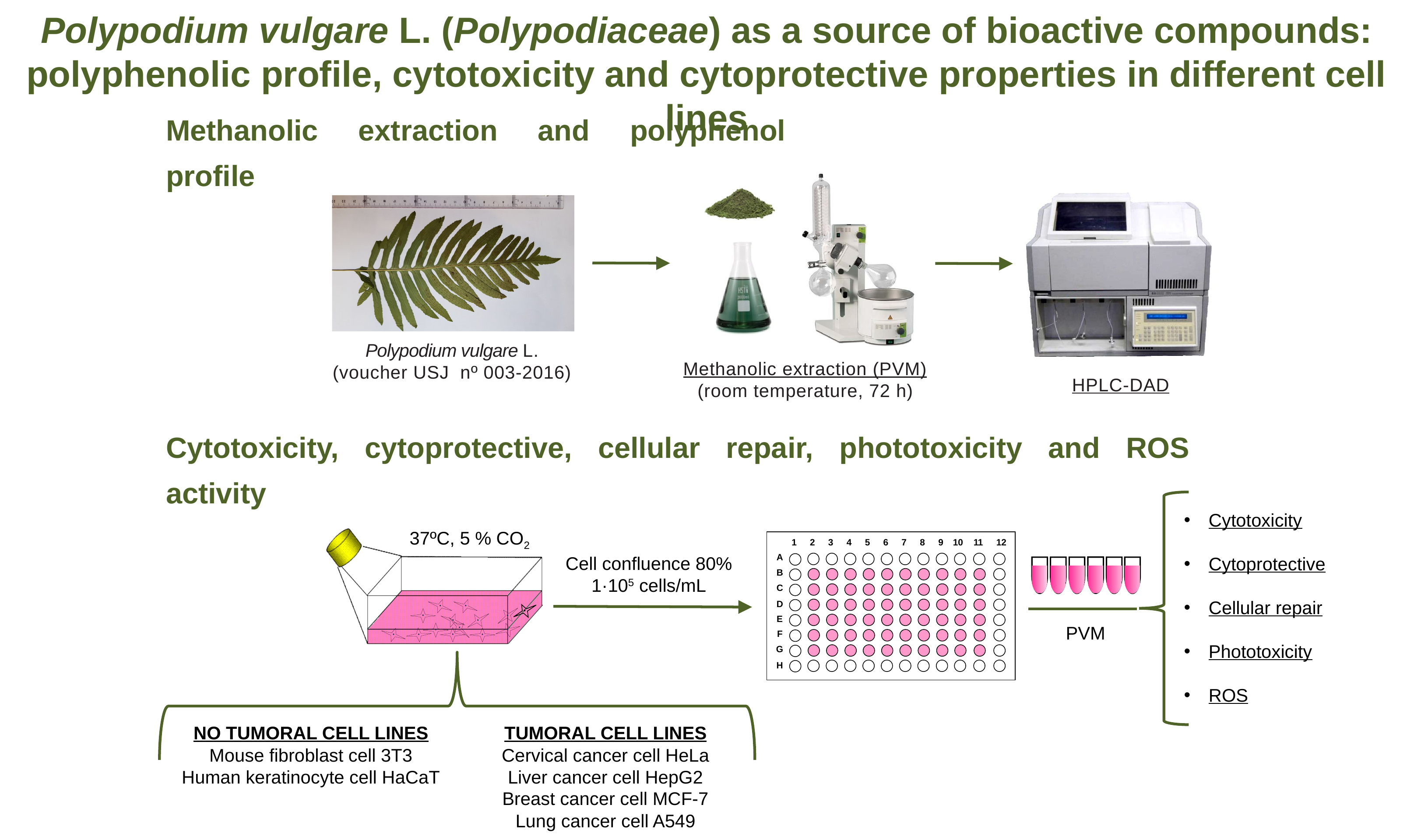

Polypodium vulgare L. (Polypodiaceae) as a source of bioactive compounds: polyphenolic profile, cytotoxicity and cytoprotective properties in different cell lines
Methanolic extraction and polyphenol profile
Polypodium vulgare L.
(voucher USJ nº 003-2016)
Methanolic extraction (PVM)
(room temperature, 72 h)
HPLC-DAD
Cytotoxicity, cytoprotective, cellular repair, phototoxicity and ROS activity
Cytotoxicity
Cytoprotective
Cellular repair
Phototoxicity
ROS
37ºC, 5 % CO2
1
2
3
4
5
6
7
9
10
11
12
8
A
B
C
D
E
F
G
H
Cell confluence 80%
1·105 cells/mL
PVM
NO TUMORAL CELL LINES
Mouse fibroblast cell 3T3
Human keratinocyte cell HaCaT
TUMORAL CELL LINES
Cervical cancer cell HeLa
Liver cancer cell HepG2
Breast cancer cell MCF-7
Lung cancer cell A549
