# Supplementary material for: Polypodium vulgare L. (Polypodiaceae) as a Source of Bioactive Compounds: Polyphenolic Profile, Cytotoxicity and Cytoprotective Properties in Different Cell Lines
Source: Front Pharmacol. 2021 Sep 16;12:727528. doi: 10.3389/fphar.2021.727528 (PMC8482143; doi:10.3389/fphar.2021.727528)
Supplement: Supplementary file 2 [file Table1.DOC]

**S1.** Calibration data, including regression equation, correlation coefficient (*R*^2^); limits of detection (LODs; µg/ml), limits of quantitation (LOQs; µg/ml)

| Compound | Regression  Equation | *R*^2^ | LOD^a^ | LOQ^b^ |
| --- | --- | --- | --- | --- |
|
| Shikimic acid | y = 25.143x + 5.0336 | 0.9999 | 0.06 | 0.20 |
| Gallic acid | y = 23.74x + 2.9068 | 0.9999 | 0.10 | 0.40 |
| 5-O-caffeoylquinic acid | y = 15.496x - 1.5918 | 0.9998 | 0.08 | 0.25 |
| 3-O-caffeoylquinic acid | y = 17.1507x - 27.5322 | 0.9930 | 0.03 | 0.10 |
| (+)-Catechin hydrate | y = 22.188x + 0.3341 | 1.0000 | 0.15 | 0.50 |
| (-)-Epicatechin | y = 21.262x + 2.3431 | 0.9999 | 0.15 | 0.50 |
| Rutin | y = 26.466x + 2.6982 | 0.9982 | 0.1 | 0.5 |
| Hyperoside | y = 32.426x - 20.7894 | 0.9993 | 0.1 | 0.5 |
| 3,5-di-O-caffeoylquinic acid | y = 18.19.x - 2.2361 | 0.9998 | 0.1 | 0.3 |

^a^LOD (limit of detection) = 3 *×* signal-to-noise (S/N) ratio. ^b^LOQ (limit of quantitation) = 10 *×* signal-to-noise (S/N) ratio.
